# Derek Jarman’s medieval blood: Queer devotion, affective medicine, and the AIDS Crisis

**DOI:** 10.1057/s41280-022-00260-0

**Published:** 2023-01-24

**Authors:** Eleanor Myerson

**Affiliations:** 1 Corpus Christi College, Cambridge, CB2 1RH UK; 2 London, UK

## Abstract

In this article I consider medieval blood imagery in the paintings, films and journals of Derek Jarman, focusing on works made between 1989–1993. Taking a transhistorical comparative approach, I analyse Jarman's images alongside his medieval sources, primarily Julian of Norwich's Revelations and Gerard of Cremona's translation of Ibn Sīnā's (Avicenna's) al-Qa'n ū n fī al-tibb (Canon of Medicine). In addition, I find my own commonalities between Jarman and the medieval, for example, juxtaposing his Queer series of paintings with MS Egerton 1821. Critics have explored the medieval as a site of historical precedent for the stigmatisation of disease, providing a reservoir of images of leprosy and plague which inform the discourse of AIDS as immoral pollution. However I follow Jarman's lead in seeking new avenues through the medieval in relation to the AIDS crisis. Refusing to accept the discourses which cast his HIV+ blood as the ultimate symbol of pollution and death, Jarman mobilised the aesthetics and imagery of medieval affective devotion as a powerful alternative. Through the deployment of these traditions, HIV+ blood becomes holy blood, the source of salvation, desire, community and healing.

*‘The colour of life departing from a broken heart is a trickle of red blood’*—Derek Jarman, Chroma (Jarman 2019b, 25)

The AIDS crisis gave renewed urgency to Derek Jarman’s lifelong search for new perspectives via the medieval past. Known during his lifetime for his avant-garde films, his multimedia paintings, his poetic journals and his queer activism, Jarman’s legacy has been kept alive through the efforts of his grassroots fanbase and his famous protégés, including Tilda Swinton. Jarman’s interdisciplinary works constantly engaged with present social and political conditions, and, crucially for this article, with premodern visual and intellectual traditions. As an artist and as an activist, Jarman evaded containment, sidestepping and stepping back into alternative ways of seeing the world. In 1987, Jarman publicly declared his HIV positive status, following which his body became a site of tabloid-fuelled discussion. In scathing queerphobic language, Jarman’s health was monitored and dissected as the subject of voyeuristic intrigue. Refusing to accept the discourses which cast his blood as the ultimate symbol of pollution and death, Jarman mobilised the aesthetics and imagery of medieval affective devotion as a powerful alternative. In this essay I explore Jarman’s deployment of medieval blood imagery in his films, paintings, and journals, focusing on works made from 1989–1993, an intensely productive period. In the medieval, Jarman found a language through which he could reconfigure a new symbolic relationship with blood, not least, his own blood.

Medieval mystical visions expressed the same basic premise as hostile discourses of HIV/AIDS: the idea that affective sympathy for another’s suffering has physical, internal consequences—that the imaginative touch of blood can alter the bodily condition of the imaginer. However, the conclusions of these discourses are evidently opposed—in the latter, both empathy and touch are rejected, while in the former, empathy and touch are eagerly, urgently desired. I contend that, in Jarman’s work, these medieval images of blood become a powerful corrective to the traumatic discourses through which his own experience was dictated and described by others.

Jarman was living and working under an intensely homophobic political climate in the UK, with the infamous Clause 28 prohibiting queer subjects from discussion in schools. In this context, visible signs of AIDS-related diseases were treated as ostracising tokens. Government-sponsored propaganda warned of, as Erica Carter wrote, the ‘danger of identification with those affected by HIV or AIDS’ (Carter and Watney 1989, 60). Both physical and emotional proximity with people with AIDS was inhibited, the horrors of AIDS being exploited to create distance between the sick and the well. An admission of sympathy could become an admission of belonging to an infected community, as if empathy could lead to transmission, as if the touch of human feeling could lead to forbidden intimacy. This paranoia has a parallel in medieval understandings of contagion. For example, Rosemary Horrox writes that it was widely believed that ‘the plague could be transmitted by imagination: that a person could be infected just by thinking about it’ (1994, 107). Beyond paranoia, medieval understandings that the plague and other diseases could be transmitted through imagination indicate the significance of the emotions in conceptions of disease and treatment.

If there is a general horror attached to the body of the person with AIDS, this horror is magnified in relation to their blood. The fear of contagion becomes overlaid with the scientifically reinforced dread of the knowledge that the AIDS virus is blood-borne. The fear of touch is now mandated—even the lover of a person with AIDS should not come into contact with their blood. Intimacy with blood becomes a lethal kink. Any eroticism attached to blood becomes taboo. By contrast, medieval mystical images of the man of sorrows ask the viewer to dwell in Christ’s blood. The desire to touch holy blood motivated arduous pilgrimages—transcontinental travels were undertaken in order to gain proximity to that precious trace of Christ’s presence. The bleeding Christ was deeply eroticised both in relic devotion and in Eucharistic practices, in which medieval monks reserved the sweet taste of God’s body for themselves, dreaming and luxuriating in the presence of His blood in their mouths.

In this essay, I draw attention to Jarman’s engagement with medieval medical traditions, a dialogue which has not previously been explored. In so doing, I build on Robert Mills’ pathbreaking art historical analysis of Derek Jarman’s medievalism (2018). Following Jarman’s affective route through the medieval, I expand on Carolyn Dinshaw’s argument ‘that amateurs can lead us outside a straitened solution to problems’ (2012, 37). Jarman’s imagination of medieval blood does not position the medieval as a site of historical precedent for the discourse of AIDS as immoral pollution, a nexus which has been comprehensively explored in professional critical discussions (Kruger 2001). Jarman gestures briefly to the role of blood in the imaginative formation of communities, writing of ‘All the old taboos of / Blood lines and blood banks / Blue blood and bad blood / Our blood and your blood’ (2019b, 96). Following Jarman, I circumvent certain significant aspects of medieval imaginations of blood, most notably medieval blood libel narratives (Rubin 1991) and the analysis of blood as a biologically essentialist signifier of ethnic and racialised identity, both of which are necessary, pressing subjects, but which fall outside the remit of this present essay*.*

‘I wrote this book in an absence of time,’ Jarman writes, in *Chroma*—‘I had to write quickly as my right eye was put out in August [...] I wrote the red on a hospital drip [...] at four in the morning, scrawled almost incoherently in the dark’ (2019b, 33). In this passage, Jarman phrases his fragmentary text as ‘a letter to you, dear reader,’ signing off, intimately ‘Derek,’ mid-book, at the conclusion of only the third chapter (2019b, 33). The pre-emptive signature acts as a reminder that in this period of his illness, Jarman worked in the knowledge that any line could be his last word. If I could, I would send this essay back to him. It is a reply to his ghost, in the year of his 80th birthday. I was born a year after Jarman died, and while I do not mean to appropriate the grief of those alive who really knew him, I belong to a queer generation whom Sarah Schulman has described as the ‘replacements for the dead’ (Schulman 2012, 6). But since their loss is irreplaceable, we merely exist in the continued moment of their mourning, learning from and inheriting the traditions of the dead without hoping to repair that wound.

## Avicenna’s blood flowing into Jarman’s imagination

In 1992, in thin, shaking handwriting that was quite unlike his usual dramatic, confident penmanship, Jarman noted down one of his overlooked influences: ‘Avicenna 980–1037 the moslem [sic] physician who wrote that like coulour [sic] cure.’^1^ The handwriting is relevant: the script evokes the hospital scene of writing, placing Jarman’s search for a new way of conceptualising healing in its urgent context of personal suffering. The note is from Jarman’s drafts for his ‘Colour book,’ eventually to be titled *Chroma.* In the cold archives in the Tate Britain (London) I find it difficult to read Jarman’s struggling hand without tears coming to my eyes. At the same time, as a medievalist, I feel a curious disciplinary pride in finding my subject here, in these conditions of recent, mortal threat. In Jarman’s continued investment in the translated authorities of medieval medicine from his hospital bed, there is a reassertion of the value of those texts. The Latin texts which shaped medieval English understandings of the body were themselves translations of Arabic works, which extended and preserved Greek texts (Gutas 1998). The title of Jarman’s colour book, *Chroma*, echoes these translated Greek traditions, as he discusses within the text: ‘In antiquity, colour (*chroma*) was considered a drug (*pharmakon*). Colour therapy’ (Jarman 2019b, 30). Alluding in his notes to Ibn Sīnā, under his Latinised name of ‘Avicenna,’ as the author of the ‘colour cure,’ Jarman remained aware of the transmission of these Greek terms through Arabic. While Jarman did not have the original Arabic sources at his disposal, he had a number of critical works in his library from which he learned of the significant influence of translated Arabic texts on medieval European culture. For example, Jarman owned a copy of F.C. Copleston’s *A History of Medieval Philosophy,* which argues that ‘We cannot...make a close study of the thought of Aquinas or of the philosophical situation in the university of Paris in the thirteenth century without becoming aware of the existence of Islamic philosophers such as Avicenna and Averroes and of Jewish thinkers such as Maimonides’ (1977, 104).^2^ References to translated medical traditions recur throughout Jarman’s journals and notebooks. He notes that ‘Of the medieval humours, choler (anger) was hot-blooded and red’ (Jarman 2018b, 32). The mode in which Jarman receives his sources is in keeping with the medieval monastic mode of reading: preserving the Latinised names for scholars, citing texts and authors but rarely providing full references. And like the medieval monks on whom he drew for inspiration, Jarman looks past Latin translations towards the original Arabic, towards the authentic, elusive source.

Blood was central to medieval medicine, as one of the four humours, the foundational principle of Galenic/Hippocratic medicine, which shaped medical practices from dietic prescriptions to bloodletting practices (Bildhauer 2006). In the published version of *Chroma,* Jarman writes: ‘Red has always embraced the hospital. The tenth-century physician, Avicenna, dressed his patients in red clothes. [...] Colour could cure. Red moved the blood. [...] If one gazed intently at red the blood would flow. This is why you should never let a person with a nose bleed see red’ (2019b, 30–1). Jarman takes a characteristically fluid approach to his source material here, absorbing quotations in the form of free indirect speech, blurring the lines between his voice and the translated voices of the past. Ibn Sīnā’s *al-Qa’nūn fī al-tibb* (Canon of Medicine) arrived in medieval England via the Latin translation of Gerard of Cremona, *Liber Canonis* (Book of the Canon), and was highly influential (Jagot 2013, 49). Gerard of Cremona (1114–1187) was a translator of Arabic texts into Latin, working in Toledo, the famed centre of Arabo-Latin scholarship. The last two lines here closely echo Oskar Gruner’s 1929 English translation of the first book of Gerard of Cremona’s Latin version of Ibn Sīnā’s *al-Qa’nūn fī al-tibb*:

Even imagination, emotional states and other agents cause the humours to move. Thus, if one were to gaze intently at something red, one would cause the sanguineous humour to move. That is why one must not let a person suffering from nose-bleeding see things of a brilliant red colour. (1973, 92)

Since Gruner’s translation is doubly removed from the original source, it is worth returning to Gerard of Cremona’s work in its medieval contexts. British Library Royal MS 12 G VI is a fourteenth-century Latin copy of the full five books of the *Liber Canonis*, rubricated and with ruled margins unsullied by marginalia—a weighty presentation-standard volume, owned by the London Blackfriars. Jarman’s reference appears in its medieval Latin version, early on in the book:

aliquas inlice species et etiam ipsemet extimatores mouent humores, verbi gratia sanguine enim mouent res intuerit rubeas qua propterea perhibemus illum cuius naribus sanguis fluit res splendorem habentis rubeum aspice (Royal MS 12 G VI, fol. 9r)

By some means or another alluring appearances and even imagination itself can move the humours: for example if someone were to look at a red object, their blood would move. For that reason we must prevent someone susceptible to nosebleeds from looking at an object with a bright red colour.^3^

This text is closely based on Ibn Sīnā’s original Arabic. In Toledo, Gerard had close access both to Arabic manuscripts and to a multilingual community of scholars, among whom he developed his Arabic language skills. The survival of this text in its manuscript form acts as a reminder of the practical mechanisms through which influence took place in the Middle Ages. The movement of ideas was a consequence of the physical mobility of individuals and communities. The arrival of the concept of humoural blood flow in medieval England witnesses the flow of books through premodern networks. Jarman’s engagement with these sources animates these already moving concepts: bringing the transnational and the translated into now transhistorical dialogues.

Ibn Sīnā’s theory of the influence of imagination on the conditions of the blood was based in his understanding of the close relationship between body and soul. Jarman was aware of these contexts, writing in his notebook for *Chroma*: ‘in jottings in the margins we find Avicenna who will have it that soul gives body to soul as body to body.’^4^ Jarman’s awareness of the partiality of his source material is evident. Nonetheless from the ‘jottings’ which he had access to, Jarman was able to participate in a meaningful dialogue with his distanced influences. Ibn Sīnā’s original Arabic—unread by Jarman—confirms Jarman’s instinctive sense of the centrality of the soul in Ibn Sīnā’s understanding of blood flow:

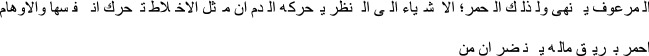
(Hameed 1982, ٥٧ [57])

And the estimative faculties of souls cause the humours to move: for example, looking at red things causes the blood to move. And therefore a person with nosebleeds should be hindered from looking at bright red objects.

For Ibn Sīnā, the faculty of estimation (*wahm*: in this passage appearing in the plural form, *al-awhām,* ) was a faculty distinct from other internal senses such as fantasy, rational imagination and memory (Yaldir 2009, 252). That is: whereas these other faculties in principle can exist independently of the body, the faculty of estimation relies on sensory perception—the presence of external stimuli on which internal images are formed. The Latin translator evidently prevaricated over the meaning of this term ‘*al-awhām*’ (estimative faculty), as did Gruner, who inserted a full extra phrase—‘imagination, emotional states and other agents’—to cover his semantic bases. In the Arabic, the faculty of ‘estimations’ is grammatically tied to ‘’—*ānfus*, souls. For Ibn Sīnā, the soul was incorporeal, immortal and capable of being separated from the body. At the same time, he contended that the imagination required the body to function. As such in this passage, Ibn Sīnā both affirms the independent influence of the soul, and demonstrates his understanding that the perceptive faculty has a direct bearing on—and depends on—the material conditions of the body.

For a visual artist such as Jarman, these ideas have an evident relevance and fascination. In attributing visual perception with the ability to affect the humours, Ibn Sīnā provides a philosophical and medicinal rationale for the centrality of the visual arts in bodily healing. As such, Ibn Sīnā’s framework gives an authoritative basis for affective reading practices in medieval texts—with a highly literal definition of ‘affect.’ That is: through this lens, looking at an image of blood can physically change the conditions of the viewer’s blood. This medically-informed definition of affective reading is especially relevant for an understanding of the late medieval devotional works to which I next turn. As Katie Walters writes: the ‘medieval habit of reading between medicine and theology [...] fosters in turn the incorporation of medical knowledge into Middle English *pastoralia* and devotional works’ (2018, 8). The pre-disciplinary quality of the Middle Ages was central to its appeal for Jarman, for example, he wrote of Roger Bacon (1214–1292) that he ‘had a mind with none of the boundaries of imagination of our time’ (2019b, 36). As such, these translated medical frameworks will be taken as essential for understanding the full meaning of the devotional texts and images which follow, both in their medieval and their modern guise. Rather than distancing myself from these medieval frameworks as the objects of my study, instead I choose to read with them, using Ibn Sīnā’s theory of the influence of the faculty of estimation (*wahm*) on the body as the basis for a historically-grounded affective reading method. I should note that this is only one route through medieval affect, which received myriad articulations and responses, secular as well as devotional (Burger and Crocker 2019).

In the context of these medical and theological works, affective reading literally occurs within the body—the reader’s visual and emotional response to texts and images is understood as altering the conditions of their blood. In light of the widespread influence of Ibn Sīnā on the development of medieval English medical imaginations, I see this understanding of affective reading as inscribed within and demanded by these medieval texts and images themselves, as well as Jarman’s responses to these texts. As I follow Jarman through medieval devotional manuscripts, I will hold the reader’s body in my mind—medieval bodies, Jarman’s body, my own body—even your body, reader, which I should warn you now, may be subject to alteration from the content which we are about to encounter.

## Jarman contemplating Julian’s blood

Medieval devotional works assert the fundamental necessity of contemplating blood (Bynum 2007). These mystical traditions explicitly combine medical and theological knowledge, drawing on the language of surgical practice and humoural medicine in order to provide a scientifically and materially grounded exegesis. Jarman drew explicitly on late medieval mystical traditions, particularly the *Revelations* of Julian of Norwich. On Tuesday 19 December 1989, he noted in his diary: ‘Read Julian of Norwich’s *Revelation of Love* to the gentle whirr of the washing machine, which HB says puts his mind at rest. [...] I sing myself to the bookshops, mind full of the Middle Ages [...] *Grete dropis of blode, in this shewing countless raindrops fall so thick no man may number them with bodily witte*’ (Jarman 2018a, 208). ‘HB’ is an abbreviation of Jarman’s pet name for his companion, Keith Collins, Hinney Beast, setting a domestic, intimate scene. Jarman’s quotation is inexact—not transcribed meticulously from a critical edition (as I will do in a moment) but brought to the reader as it has been filtered through Jarman’s mind—as Jarman sung the lines to himself as he walked through central London. The recitative quality of Jarman’s use of the text conveys his intimate engagement with the book, a ritualistic interiority that reflects his deep absorption of both aesthetics and practices of medieval devotion. Julian’s original text reads as follows:

The great drops of blood fell down from under the garland like pellets, seeming as if it had come out of the veins; [...] The abundance is like the drops of water that fall from the eaves after a great shower of rain, which fall so thick that no one may count them with their bodily wit. (Julian 2015, 39)

Julian’s language here invokes a range of images grounded in everyday material experience. The raindrops are imagined not generally falling from clouds, but specifically running from the ‘eves’ - the jutting edges of roofs, sketching the scene of medieval Norwich as viewed from Julian’s anchoritic cell. The same highly specific materiality is visible in Julian’s surgical image of blood rushing from the veins in Christ’s forehead. The description evokes the figure of ‘homo venorum’ [vein man] found illustrating medieval bloodletting manuals, as in the *Guild Book of the Barber-Surgeons of York*. In this illustration, the veins of the body are labelled separately, with the vein in the forehead specified as good to open for ‘frensy and pimples in the face and also for haemorrhoids and for madness,’ while the vein in the temple is for ‘aching and throbbing in the head’ (Egerton 2572, fol. 50r). In specifying that Christ’s blood seems to come from the veins, Julian references the medieval medical orthodoxy of the understanding of the body and means of treatment. Orthodoxy here is swiftly made transcendent through the transition from corporeal to nebulous form, the veins of the forehead ascending and vaporising into a rain cloud with the gushing of cupped blood becoming the fertilising shower of rain.

In *The Garden* (1990)*,* Jarman takes the viewer through a dream vision version of the life of Christ. In the autobiographical frame of the film*,* we see Jarman at his desk, writing or dreaming. Placing himself within the narrative, Jarman situates his retelling of the Passion in a contemporary context, setting Christ’s blood in relation to his own. *The Garden* was filmed at Prospect Cottage, Dungeness (Kent), the former fisherman’s cottage which Jarman purchased following his initial HIV diagnosis. In early draft scripts, *The Garden* was titled *Borrowed Time*: what Jarman felt he was living on (Peake, 397). In Jarman’s version, the Passion becomes a tragic romance—the figure of Christ is split into two, a couple, one lover played by Keith Collins, the other by Johnny Mills. Previously, Collins has appeared in the role of silent companion while Jarman studied Julian’s theology; here he becomes the protagonist of Julian’s visions. The men are bound to the crucifix and whipped. The camera zooms in on the blood soaking through their white shirts, the blood spreading out across their backs, as well as beading on their foreheads. Niall Richardson has argued that Jarman’s deployment of blood imagery is his means of evoking the experience of AIDS, while avoiding the stereotypical depictions of AIDS in film and media (2009, 189–92). For Richardson, Jarman’s focus on blood, in the context of the AIDS crisis, immediately invokes the threat of contagion from the blood-borne virus. While I agree with Richardson that Jarman’s blood imagery derives from the desire to represent AIDS through an alternative visual language, for me Jarman’s blood imagery expresses not simply an alternative visual language for the representation of AIDS, but specifically offers an alternative visual language which reconfigures queer blood in the context of AIDS. In addition to focusing on the bloody bodies of his two Christs, Jarman presents the Passion against the backdrop of a blood-red sea, cutting back to a full screen of red liquid directly after the Last Supper. In focusing on this expanse of red colour, explicitly juxtaposed with the blood of the Passion, Jarman deploys the visual language of affective piety expressed in Julian’s *Revelations*. Julian’s rain shower of blood is even magnified, deepened, as the ocean itself washes blood onto the Kentish stones. Rather than invoking contagion, the blood washed in by the ocean becomes the cleansing blood of the Passion. Rather than expressing the imminence of death in a straightforward way, this devotional blood is salvific, transcendent and above all desirable to touch. The suffering of the men, expressed through their profuse bleeding, demands that we approach them affectively.

Jarman’s filmic representation of Collins’ blood as salvific, through the self-conscious casting of his partner as one half of Jesus, has an echo in Jarman’s life-writing. In a draft for *Chroma,* Jarman writes: ‘I am sitting at Barts with HB, waiting to be connected to the drip of DHPG Gam-cyclovir. HB offers me his blood, “it will kill everything and be romantic,” he says. The drip trills like a canary and gets going. I watch the blood drain back into my arm.’^5^ Undergoing treatment for cytomegalovirus (CMV), the AIDS-related disease that was attacking Jarman’s retinas, the medical significance of blood is paramount here, with the drip attached to Jarman’s body. Collins’ characterisation of his blood as curative is presented as an act of devotion akin to the role of blood in his performance of the Passion. Collins’ offer of blood is presented as a self-sacrifice motivated by love. Jarman trimmed the description down for the final text of *Chroma* and the script of *Blue*: the line becomes more simply: ‘HB offers me his blood. / It will kill everything, he says’ (Jarman 2019b, 86). Collins’ own speech—recorded in Jarman’s hand—emphasises the devotional quality of his offer, a metaphorical expression of his highly non-metaphorical desire to cure his partner from the mortal threat which hung over them both. Collins as actor, companion, and carer frames Jarman’s understanding of holy blood. While Collins’ blood cannot remove the HIV virus from his body, the act of sitting beside him in the hospital appears as an embodiment of the daily realities of Collins’ care that Jarman experienced.

In Julian’s *Revelations,* the image of Christ as a physician is recurrent. She writes: ‘And then it came to my mind that God has made plentiful waters on earth [...] but still he prefers that we take his blessed blood, full intimately, to wash us from sin, for there is no licour that is made that he likes so well to give us’ (Julian 2015, 49). As in Keith Collins’ offer of his curative blood to Jarman, Julian’s theology of Christ’s sacrificial blood is an expression of Jesus’ love: the act follows his ‘liking.’ Simultaneously romantic and medicinal, Julian invokes the idea of Christ’s blood as a cleansing ‘licour’—employing the term typically used in medical surgical vocabulary. Extending this conceit, Julian writes: ‘For by these medicines every soul should be healed. Though he be healed, his wounds are seen before God, not as wounds, but as worships’ (Julian 2015, 89). The idea of Christ’s wounds as medicinal was reflected in material cultures of medical-devotional objects, such as the late fifteenth-century Coventry Ring (British Museum no. AF.897), which depicts Christ’s wounds in red enamel, alongside the inscription ‘crux et passio Cri sunt medicina michi’ (the Cross and the passion of Christ are my medicines), as well as the pseudo-Hebrew charm word ‘ananyzapta.’ This medical charm ring is at once an intimate expression of attachment to Christ and an integration of that devotional practice into understandings of medical cure. The explicit conjunction creates both a medically inflected affective tradition and an affective medical tradition.

In one of Jarman’s drafts for *Chroma—*here titled ‘A Riot of Colour’—he writes of ‘The red Christ of sacrifice. The son who is the red of the Trinity. The blood of whose sacrifice burns in a thousand red lights in the gloom of churches. Christ healed the sick. Each victory of the red cells brings death... for the virus is red. [...] Red plague cross.’^6^ In this passage, Jarman moves through a series of medieval-style juxtapositions of medical and religious ideas. The image of Christ as a physician—which was removed for the published edition—directly follows the invocation of Christ’s blood as sacrificial. The symbolism of red extends into Jarman’s present with the red ribbons worn at AIDS funerals and the red blood cells’ replacement of diminishing white blood cells in the body of a person with AIDS. Finally, Jarman turns to the symbol of medieval plague, the red cross marked on the door of an infected household. Jarman expresses his medievalism through associative thinking, showing the integration of these texts and histories into his imagination, his intimate relation with the past and the suffering of the past. Julian of Norwich lived through the first surge and the subsequent decades of outbreaks of plague during the Black Death, experiencing the visions on which she based her *Revelations* in 1373. As J.D. Fulloon notes, 56% of the population of Norwich died of plague in the first wave: before 1349, Norwich was the second largest city in England, and never recovered its population density (2021, 463–4). When Jarman draws on Julian’s affective mystical visions of Christ’s blood, he invokes a tradition that was steeped in medical imagery, and that was fundamentally rooted in the observation and experience of human disease. The historic medical contexts of medieval texts are an explicit presence, as vividly evoked in devotional texts as Jarman’s contemporary medical contexts are evoked in his own artistic responses. In both medieval and modern works, where blood functions as a metaphor, it is a metaphor based on direct, proximate observation and experience of the realities of blood and illness.

## Good blood and corrupt blood

Julian’s use of medical imagery is no unique incidence, but part of a pattern in late medieval vernacular devotional works on the Passion, which combined medical and spiritual discourses (Lucas 2020, 3). The Middle English *Doctrine of the Hert* survives in four fifteenth-century manuscripts, two of which (Cambridge, Fitzwilliam Museum, MS McClean 132 and Oxford, Bodleian Library, MS Laud Misc. 330) appear to have been compiled specifically for enclosed women, anchoritic readers (Whitehead 2009, xxi). The English *Doctrine* is based on the mid thirteenth-century Latin *De doctrina cordis,* of which 208 extant manuscripts survive across Europe, which indicates a significant transnational readership (Whitehead 2009, xi). All of the manuscripts of the English *Doctrine* directly address a readership of women, varying only in terms of whether that audience is implied as being comprised of laywomen, nuns, or anchorites (Whitehead 2009, xxii). This text reinforces the conjunction of bloodletting and devotional imagery:

Sister [...] let out the corrupt blood of sin by the vein of your mouth and keep in your good blood [...] Marvel not though I call the mouth a vein, and sin corrupt blood, for so I find written in the proverbs of Solomon: *Vena vite os iusti,* ‘The mouth of a righteous man is a vein of life.’ When is your mouth, sister, a vein of life? Truly, when you put out from your heart the corrupt blood of sin by the lancet [‘lawncete’] of sorrow and contrition. (Whitehead 2009, 7)

The image is intended as an injunction for the reader to attend confession. The practice with salvific implications for the soul is here expressed in medical terms. The ‘lawncete’ extends the metaphor through reference to the surgical instrument employed for bloodletting. Confounding modern assumptions, this text reveals a scene of late medieval women’s devotion in which knowledge of Latin scripture is not assumed, but knowledge of surgical practice is. A highly developed understanding of medicine is assumed on the part of the reader—the addressed ‘sister’ of the Middle English treatise—who is not assumed to be literate in Latin scripture, but is assumed to understand these scientific references and to be able to use them to enhance her readings of theology. These medieval mystical metaphors of bloodletting draw on detailed knowledge of contemporary surgery, employed in conditions of urgent need.

Both Julian’s *Revelations* and the *Doctrine of the Hert* situate Christ’s blood in a range of medical contexts. The hot temperature of the blood specified in Julian’s vision above, is given elaboration in the *Doctrine of the Hert*: blood ‘comes out so boilingly from the heart of our lord, made hot by the fire of love abounding in his breast, that it both cleanses the heart from sin, and also scalds the devil’ (Whitehead 2009, 37). Temperature assumes a crucial role in surgical practice, as expressed here. The cleansing action of the blood depends on the heat with which the fluid meets the organ. The *Doctrine*-author continues:

our lord Jesus [...] has not only ordained for you a cleansing bath of water and blood in your baptism, but also a healing bath of sweat and tears, that you should both wash yourself in and become whole. Into this path you must often enter, for it is medicinal. For to enter into this bath is nothing else but to drown your affection and your thoughts in Christ’s passion, considering both the shedding of his blood and water, and also the sweating and the weeping of his body. (Whitehead 2009, 37)

The ‘medicinable’ bath of sweat evokes the surgical practice of sweating a patient in preparation for bloodletting (Citrome 2016). The bath is both an expression of affective excess, and an allusion to a specific medical treatment, appropriately to the pre-existing surgical context of bloodletting. As such this image combines awareness of a number of different medical techniques, which were used in combination in medieval surgical practices: the use of bloodletting, the use of boiling liquid for the cleansing of wounds, and the use of medicinal baths for treatment associated with bloodletting.

In addition to the understanding of medieval medical blood gained through Julian’s *Revelations,* Jarman owned an abridged edition of John Trevisa’s fourteenth-century Middle English translation of Bartholomaeus Anglicus’ encyclopaedic work *On the Properties of Things,* in selections made by Robert Steele in 1905. In this book of *Mediaeval Lore from Bartholomew Anglicus, *Jarman could read that:

a vein is the bearer and carrier of blood, keeper and warden of the life of beasts. And containeth in itself the four bloody humours clean and pure [...]. Also a vein is messager of health and of sickness [...] if a vein be corrupt, and containeth corrupt blood, it corrupteth and infecteth all the body. (Steele 2004, 81–2)

This understanding of the veins underpinned the practice of bloodletting, and shaped Julian’s imagination of the Passion. The medieval surgeon John of Burgundy’s treatise on bloodletting situates this practice in the devastating context of the Second Plague Pandemic: ‘A noble tretise made of a noble physician Johne of Burgus for medicine against the Pestilence’ (Egerton 2572, fol. 67r). The treatise appears within the above-mentioned *Guild Book of the Barber-Surgeons of York*, the guild context emphasising that this was a text designed for practical application. The manuscript acts as a witness to the historical use of bloodletting in a plague environment. As such, encountering medieval medicine in its surviving material contexts enables affective engagement with the experience of bloodletting patients, to which the mediated excerpts in Steele’s edition of *Medieval Lore* gestures. The first part of John of Burgundy’s treatise is devoted to the role of diet in avoiding the plague, for instance, the author recommends pickled fruits and vinegared drinks. The second part addresses the use of bloodletting in treating the ‘boche’—the bodily swellings which remain such a notorious symbol of medieval plague, appearing in the armpits and across the body. The author explains the rationale for the use of bloodletting: ‘in each man there are three principal parts of his body, the heart, the liver and the brain and each one of these has his place where he may put out his superfluities and clean him. The heart is cleansed [by opening the vein] under the arms in the armpits, the liver between the thighs and the body, the heart under your ears and under your throat’ (Egerton 2572, fol. 67v). The surgeon’s task is therefore to locate the cause of the disease—in the heart, the liver, or the brain—and to apply cupping glasses for bloodletting to the corresponding part of the body as required to cleanse that diseased location—respectively, at the armhole, the inner thigh, or under the ear or throat. The author warns:

if 23 hours pass before you bleed [the patient] the matter is gathered and hardened and will not pass out of the veins, nevertheless if you can bleed it may not harm, but it is not certain to help you. For if the matter is gathered below either armpit, it comes out of the heart, so bleed on the heart vein that is called *cardiaca* [...] or else follows two harms: [...] the good blood that is not poisoned or corrupted will not be drawn out and the blood that is corrupted and poisoned will pass to the heart and bring a man to his death (Egerton 2572, fol. 68r)

The urgency of this observed reality that the corrupt blood, if not drawn out, will pass to the heart and cause a man to die underpins the mystical imagination of the corrupt blood within the body, requiring bloodletting through the vein of the mouth. A detailed understanding of this medical context informs the metaphor of confession, which underlines the religious understanding of confession as a matter of life and death. An awareness of the limitations of these practices is written into this plague treatise. This text does not speak from a grandiose position of certainty, but from a position of authority tempered by the understanding of the frailty of the human condition, over which the doctor does not assume final control, but defers to a higher power: ‘And [if a person] rules themself after the teaching of this treatise through the grace of god he shall be helped from his sickness’ (Egerton 2572, fol. 69r). The text does not dismiss medicine in favour of prayer alone, but rather attributes the effectiveness of surgery—when it is found to be effective—to God’s grace. An integrated medico-religious sensibility is therefore present in both surgical and devotional genres, which together provide a vivid picture of the role of bloodletting in medieval culture in a time of plague. The appearance of the bloodletting metaphor in medieval devotional works should therefore be read with this historical context in mind as a metaphor drawn from a surgically informed perspective on the realities of human precarity. John of Burgundy’s treatise asserts not the total effectiveness of bloodletting, but the necessity of continuing to attempt healing in the context of the known limitations of the practices available. The medieval surgeon is not characterised as working from an omniscient position but neither is the surgeon acting from a position of naivety and superstition, as modern stereotypes of the medieval contend. The vocabulary of bloodletting is the canonical received vocabulary of translated transnational medicine. In order to learn from medieval imaginations, we must approach these texts as Jarman did: from up close and personal, from an affective position which allows for transhistorical commonality rather than distance (Turner 2016).

As an AIDS patient in the 1990s, Jarman lived with the constant awareness of the medical and societal view of his own blood as polluted. In *Chroma,* he writes:

The side effects of DHPG, the drug for which I have to come into hospital to be dripped twice a day, are: low white blood cell count, [...] low platelet count which may increase the risk of bleeding, low red blood cell count (anaemia), [...] high blood pressure (hypertension), low blood pressure (hypotension), [...] bleeding from the stomach or intestine (intestinal haemorrhage), [...] increased number of one type of white blood cell, low blood sugar, [...] blood in the urine, [...] increased blood urea [...]. If you are concerned about any of the above side effects or if you would like any further information, please ask your doctor. (Jarman 2019b, 92–3)

The above list is excerpted to reflect only those side effects which directly pertain to blood—other potential consequences of DHPG range from anorexia to ataxia, coma, psychosis, infertility, potential carcinogen, swellings. The text subsequently became part of the script for Jarman’s experimental film *Blue* (1993). The screen is a constant shade of International Klein Blue throughout the film, the voiceover received simultaneously with the pulsating monochrome screen. Jarman invites the viewer to stay in the blue flashes of his visual disorientation. At a screening of *Blue,* the light washes over the audience, filling the room, so that the room becomes a body and the screen an opaque eye through which we see only one colour. Jarman’s selected colour evokes the painter Yves Klein’s mystical abstraction, which itself drew on traditions of saint devotion. In the context of Jarman’s readings of medieval medical colour theory, the visuals of *Blue* assume increased significance, setting the concept of curative colour against his experience of medical treatment. I am not alone in flinching as I listen to these lines, my stomach contracting involuntarily and my shoulders tensing: Hallas has described how *Blue* engenders an ‘embodied spectatorship’ (Hallas 2009, 231). The extremity of the list with its catalogue of what seems like the full range of conditions of human suffering, heightens our sense of the banality of the final line and the bureaucratic formalities of modern clinical medicine. The conjunction ‘if’ appears as an aberration. It is unimaginable that these diseases would spark no concern, as if the human condition itself could be dismissed as unworthy of comment—but the phrasing is deliberately distancing, immunising the constructed doctor against the emotional impact of the prescribed treatment. Rather than the affective response experienced by Jarman’s readers or listeners, the voice of this line is notably disaffected; evidencing a detachment which, following Foucault, has been identified as a fundamental quality in the creation of the category of the clinic (Diedrich 2016, 84). It should be clarified that Jarman does not elide healthcare workers with the structural framework within which they operate: he dedicates the film to his nurses, alongside ‘HB, and all true lovers.’ At the same time, Jarman establishes a sharp contrast between the affective, quasi-mystical discourse of the medieval colour cure, and the objective figure of the modern neutral clinical persona.

## The healing spectacle of blood

In the early 1990s, Jarman began work on a series of paintings known as his *Queer* series, after the title of the 1992 solo show at Manchester Art Gallery at which these works were first displayed. A sequence of striking, large-scale works, these paintings combine the aesthetics of medieval blood devotion, with caustic engagement with contemporary media discourses of AIDS. To me, these works are among Jarman’s most explicit invocations of the established visual traditions of medieval affective piety. In keeping with these traditions, Jarman was invested in the visual articulacy of blood. He records asking his doctor whether he could paint using his blood: ‘he was enthusiastic and he said he’d find out if it could be sterilised’ (Jarman 2018b, 72). In the absence of following records, the assumption must be that the project was thwarted at the clinical stage, Jarman’s blood deemed too contagious for safe deployment in artistic endeavour. These paintings therefore assume increased significance, as surrogates for Jarman’s own blood as denied material, the red paint acting as a representational substance with direct correspondence to the absent bodily fluid.

**Figure 1:**
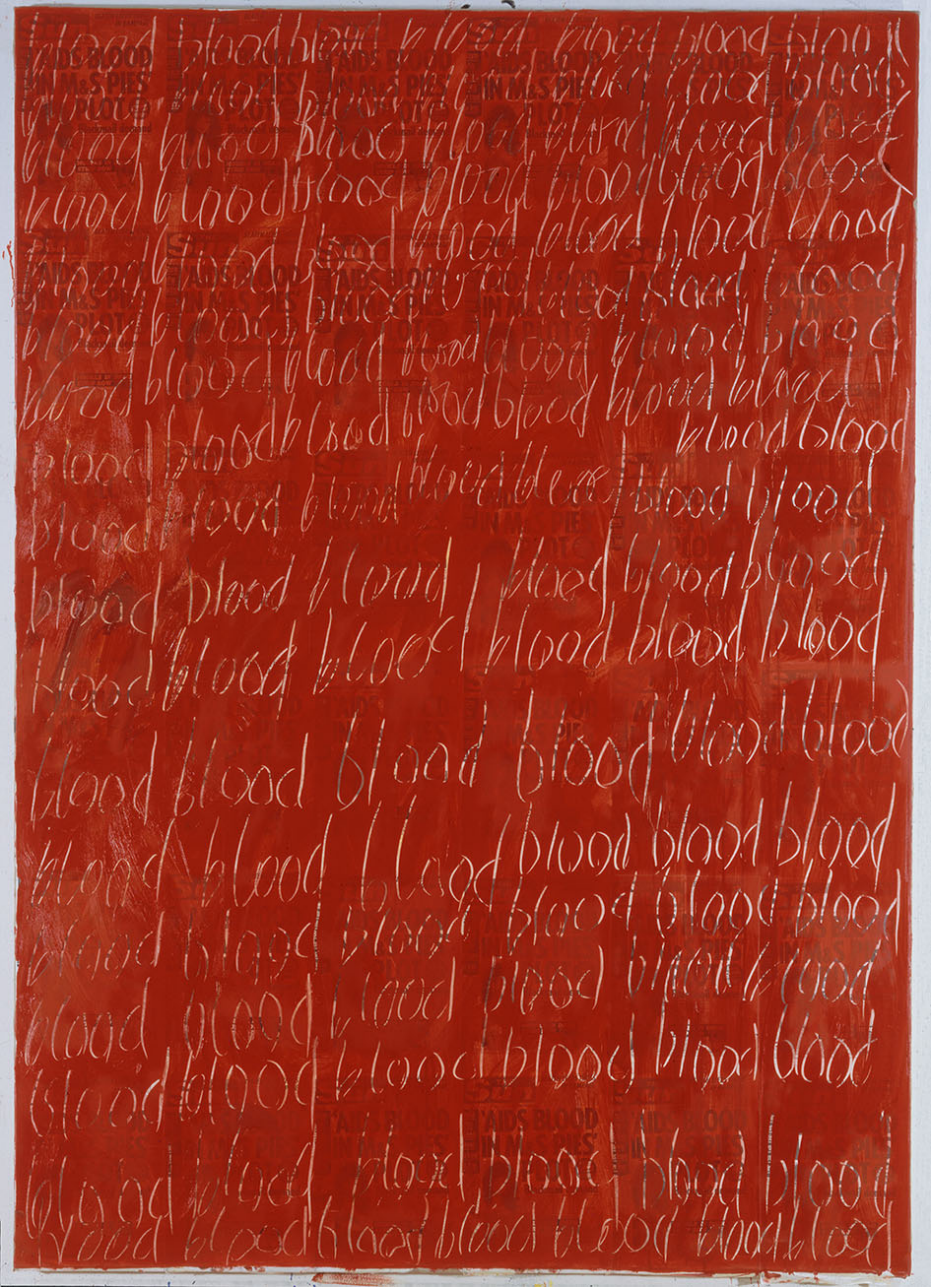
*Blood,* 1992, oil and photocopy on canvas, 251.4 x 179 cm. Manchester Art Gallery, Manchester. Detail. Courtesy Keith Collins Will Trust

In *Blood* (1992), Jarman repeatedly scrawls the word in the surface of red paint, through which a headline is legible: ‘AIDS BLOOD IN M&S PIES PLOT’ [Figure 1]. This frontpage casts AIDS blood as an insidious, invasive presence inside two of the most normalised and domesticated of locations in Britain: Marks & Spencer, the archetypal middle-class suburban supermarket, and the pie, the archetypal English comfort food. The M&S pie arrives in the imagination with its context of consumption unspoken but unavoidable, that is: the Conservative family unit. ‘Even here!’ the headline tells its reader—even your domestic dinners are not safe from AIDS! The headline withdraws and recoils from the contaminating touch of blood. Jarman made these works with the aid of assistants (Piers Clemmet and Karl Lydon), as CMV hastened the deterioration of his eyesight (Peake 1999, 521). In demanding that our attention focus on the realities of blood, he also demands a renewed affective attention to his own specific trauma, the body which he felt trapped within as he describes his mind like ‘a naked lightbulb in a darkened ruined room’ (Jarman 2018b, 193). In these works, Jarman invites us to enter the room and look at the world from within that perspective, to look out at the UK tabloids through the haze of blood which overlaid Jarman’s. On the surface, then, *Blood* is a painting which returns us to the alienating context of the early 1990s. The overlaid expanse of red colour evidences the influence of Abstract Expressionism, and, as Robert Mills has noted, the repeated handwritten motif has echoes in aspects of Andy Warhol’s silkscreen prints and Cy Twombly’s gestural scripts (2022). Although these associations remain relevant, the blood in Jarman’s work exceeds these referents: through allusion to the visual language of medieval blood devotion, Jarman’s red becomes representational, not purely abstract.

**Figure 2:**
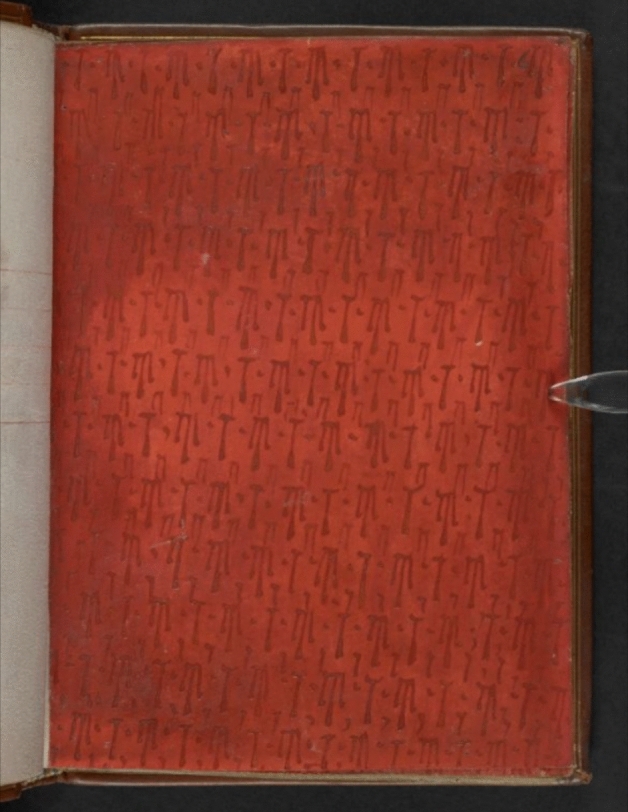
British Library Board. MS Egerton 1821, c. 1525–1538, Parchment, 180 x 130mm. British Library, London, fol. 6v. British Library Digitised Manuscripts Online

The medieval affective language of blood, which Jarman encountered in textual form through Julian of Norwich’s *Revelations*, found visual expression in a wide array of devotional objects. For example, in Julian’s fourth vision of Christ’s Passion, she sees that ‘so plenteously the hot blood ran out that there was neither seen skin nor wound, but as it were all blood [...] And this was so plenteous to my sight that methought, if it had been so in nature and in substance at that time, it should have made the bed all one blood’ (Julian 2015, 49). Julian’s striking image of the Passion as expressed in blood alone is given visual expression in a number of artefacts; one close comparison is found in British Library MS Egerton 1821, an early sixteenth-century book which reflects late medieval devotional traditions. The item is digitised, but the computer screen enlarges and flattens the object. The affective impact of its size is experienced differently in person, in the touch of the hand to the pores of the page: a page which was once stroked so insistently by medieval hands that the surface of the skin is worn away in places. A sequence of five full pages is washed with nothing but red ink, and darker red blood drops slightly elevated on the sheet [Figure 2]. Although to the modern eye these sheets are strikingly abstract, the red here is figurative, depicting the non-substitutable blood of Christ. The sensationalism with which Egerton 1821 approaches the Passion as seen in the scale of blood, and the graphic representation provides the reader with a powerful tool for affective devotion. In Julian’s vision, blood obscures all other signs, both inside and outside the scene of the Passion—overrunning skin, wound, and bed alike. The pure representation of blood in Egerton 1821 therefore provides an actualisation of Julian’s speculative phrase ‘as it were’—requiring the reader to turn over five full sheets of blood before finally reading a visual representation of Christ’s Passion in the woodcut pasted over another red page, with yet more dark red drops painted over his body (Egerton 1821, fol. 8v). Here, the reproductive mechanism of the printing press is surpassed by the manual reproduction of the illustrator, who has painstakingly repeated drop after drop. Furthermore, the two shades of red used in Egerton 1821 reflect the shades specified in Julian’s first vision of Christ’s blood: ‘in the coming out it was brown red, for the blood was full thick, and in the spreading about it was bright red’ (Julian 2015, 39). The colour of both ‘brown red’ and ‘bright red’ hues remain luridly clear on the medieval page: the insistent materiality of the colour is at the forefront of the reader’s experience.

The act of looking at Christ’s blood, which was endowed with a spiritually healing effect on the contemplative viewer’s soul, can also be seen as having a corporeal impact on the contemplative viewer’s blood. As seen in the devotional work, *Doctrine of the Hert*, medical orthodoxies reached a wide medieval audience, far beyond Latinate monastic and medical professionals. Although there is no direct allusion in Egerton 1821 to the humoural impact of visual perception of colour, as expressed in Ibn Sīnā’s *al-Qa’nūn fī al-tibb*, these red pages assert their bloody impact, encouraging readerly engagement through contemplation. The contemplative engagement with colour, which this manuscript demands, is closely aligned with the idea previously explored, that a representation of the colour red can have an internal bodily effect. Seen through a medieval medical lens, there is a sense that these representations of blood will directly impact the reader’s body in two ways at once: spiritually cleansing and physically animating, via the reader’s faculty of estimation (*wahm*)*.* Text and body are placed in active relation. Considering these pages with the reader’s body in mind heightens the importance of the repetition of the colour—the scale of the red blood drops is not only given metaphoric significance but also allowed to assume medically-derived potency. The reader’s connection with the suffering of Christ is facilitated by these blood pages in internal, corporeal reactive form.

In the painting *Blood,* Jarman’s repetition of the word and the excess of red paint recall the scribal action and aesthetics embodied in Egerton 1821. While I do not suggest the direct influence of this specific manuscript on Jarman’s painting, to me the close overlap between these works speaks to Jarman’s in-depth engagement with medieval visual affective traditions. The illuminator of Egerton 1821 draws blood drops over pasted-in woodcuts. Jarman’s layers of red paint cover the ‘skin’ of tabloid sheets. Both objects invite the viewer to focus on their surface: the medieval reader rubbing away to the point of tearing the devotional book, the modern viewer drawn to the surface of the painting through the thickness of the oils, into which Jarman has scratched his text. Repetition is a meditative device. In both, the viewer is invited to stay with the contemplation of blood, of pain, affectively sharing in suffering rather than looking away. The juxtaposition with headline from the British tabloid, *The Sun*, offsets this affective practice against its alternative: an obsessive paranoia. Through the invocation of the aesthetics and meditative repetition of affective piety, Jarman’s painting requires us instead to approach, to linger, to imagine the taste of blood not with horror, as part of a grotesque fantasy of blood-laced pastry, but as a devotional practice.

## Queer devotion

**Figure 3:**
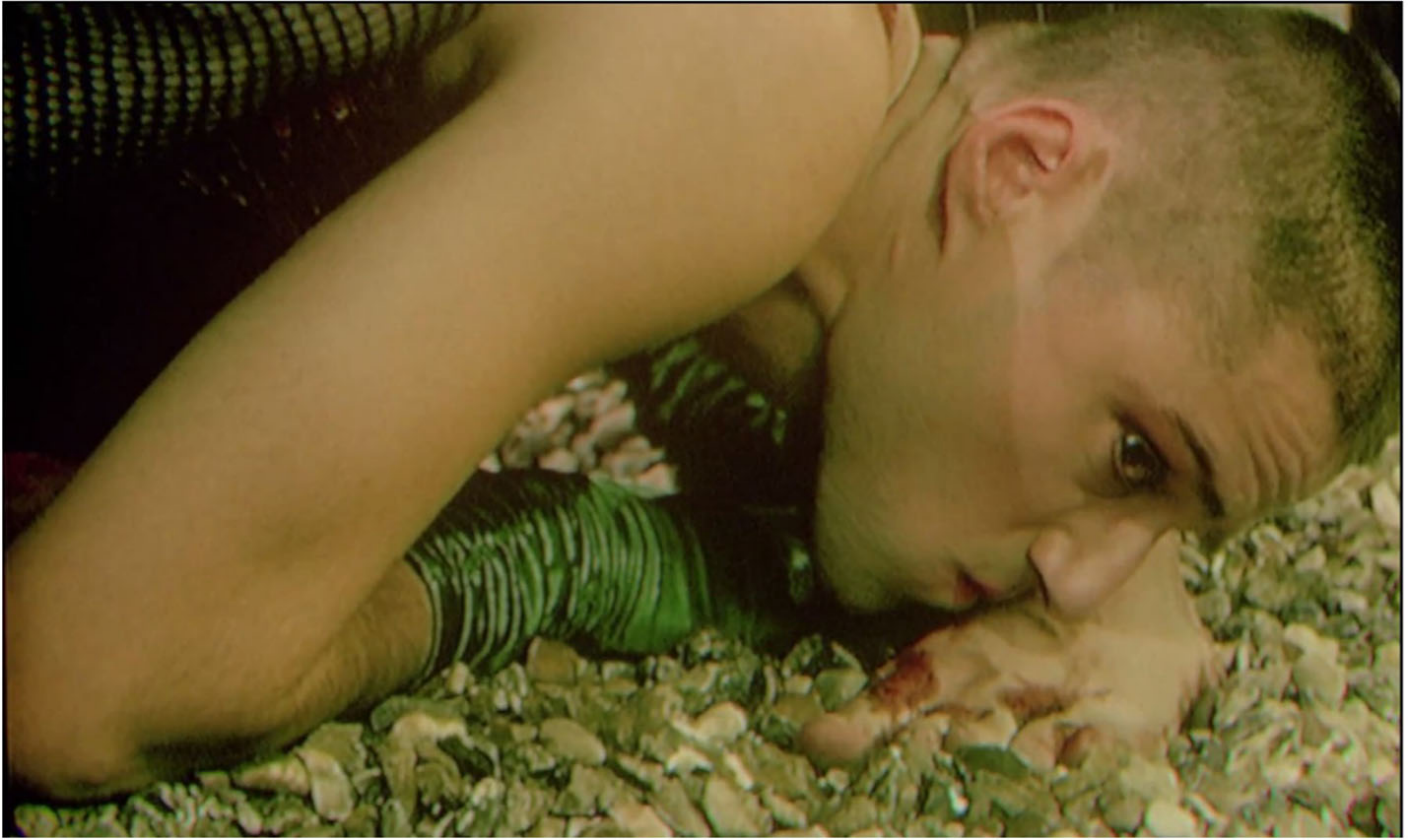
*The Garden,* dir. Derek Jarman (1990). Screenshot from DVD

Jarman’s film, *The Garden,* presents not only an emblem of queer Passion, but also an inscribed devotional response. As the Crucifixion approaches, the men bear their crosses together, stripped to the waist and already bleeding. Mary Magdalen appears in dark pink sequins and green satin evening gloves, but her emotion is irrepressible, she removes her wig in grief and anger and kneels to kiss the feet of the condemned boys. The camera lingers on her shaven head, her eyes intense with emotion [Figure 3]. In this figure, Jarman offers us a personification of affective devotion. This representation of Mary Magdalen recalls Jarman’s own commitment and delight in ‘radical drag,’ which he saw, somewhat problematically, as a tribute to the trans heroines of the Stonewall riot. Jarman even won Alternative Miss World 1973, a vision in pearls as Miss Crepe Suzette (Jarman 2019a, 69). In order to grieve the Passion in Jarman’s retelling, the viewer must assume the gaze of this queer mourner. The scene evokes the rage and trauma of mourning during the AIDS crisis, but with a crucial difference. Mary Magdalen kisses the bleeding feet of the condemned couple, her lips grazing their open wounds. Rather than the fluid being forbidden to touch, in this restaging of the Passion the holy blood is tangible. At this point in the narrative, there is no hint of the biblical injunction *noli me tangere* (don’t touch me) given by Jesus to Mary Magdalen after the Resurrection (John 20:17). AIDS funerals were too often scarred by this same injunction. At Jarman’s own funeral, Collins had to argue furiously with undertakers who wanted Jarman’s coffin sealed, in contradiction of Jarman’s wishes to have the casket open until the burial (Peake 1999, 533). Jarman’s crucifixion scene dwells on queer communality and touch, offering a vision of unmediated grieving.

Jarman’s vision of Mary Magdalen does not only draw on his contemporary scenes of drag balls and queer funerals, but on the genderqueer imagination of medieval affective devotion (Spencer-Hall and Gutt 2021). Julian of Norwich presents a fluid sense of Jesus’s gender when she writes: ‘meekly make we our lament to our beloved mother, and he shall all besprinkle us in his precious blood’ (Julian 2015, 131). In *Modern Nature*, Jarman alludes to this passage, noting: ‘Julian says *It is today domysday with me, oh dereworthy moder*’ (Jarman 2018a, 208)*.* As previously, Jarman’s quotation is inexact, not transcribed but recalled from his own memory of the text. The scene to which Jarman alludes here is that of Julian’s apparent deathbed. Having prayed to become sick, she addresses her worldly companions on the assumption of her immanent passing. Jarman interpolates the phrase ‘dereworthy moder’ (beloved mother) replacing Julian’s companions with Jesus, the subject of her visions. Jesus’s blood remains a constant presence in Julian’s visions. It is through his ‘dereworthy blood’ (Julian 2015, 129)—his ‘beloved blood’— that he expresses his maternal nature, that he becomes a mother (Bynum 1982). The image is extended: ‘our tender mother Jesus, he may lead us intimately into his blessed breast by his sweet open side’ (Julian 2015, 126). Julian’s representation of the wound as a breast provides a de-essentialised imagination of gender, which remains rooted in an understanding of Jesus’s physical body, but which recasts and re-genders that body through language. As Jesus hovers between genders, the reader’s sense of what a breast may be, of what a mother’s body might look like, expands.

**Figure 4:**
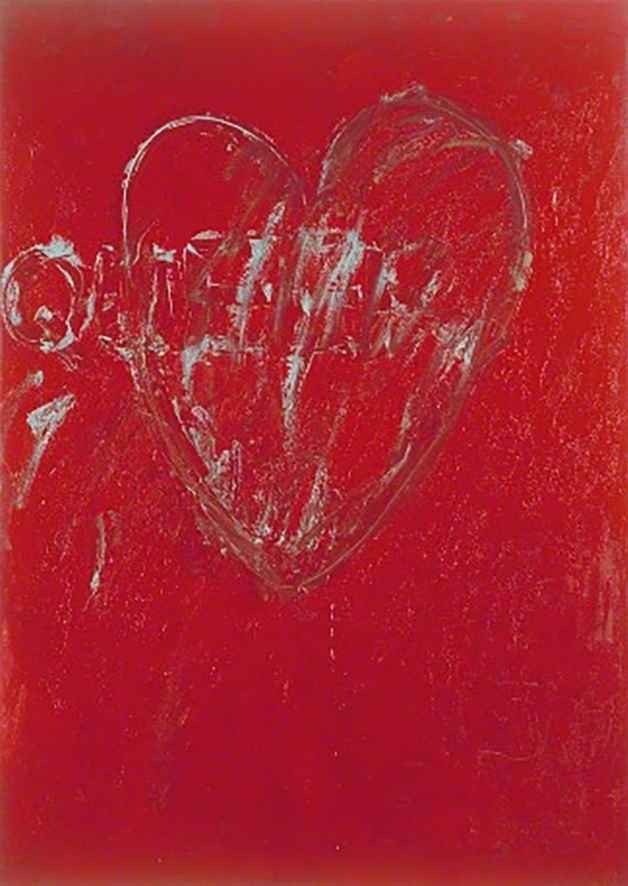
*Queer,* 1992, oil on canvas, 251.4 x 179 cm. Manchester Art Gallery, Manchester. Courtesy Keith Collins Will Trust

**Figure 5:**
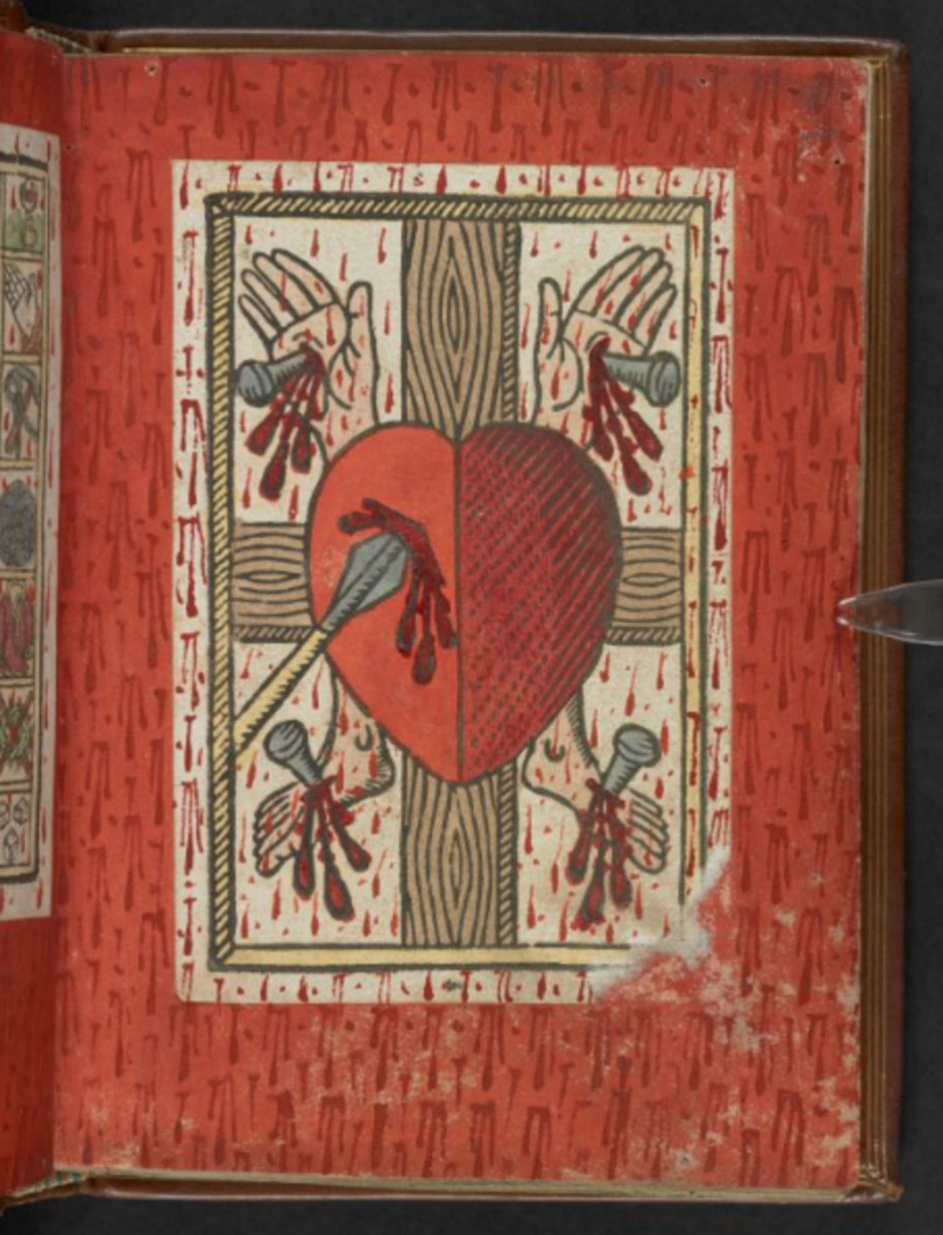
© British Library Board. MS Egerton 1821, fol. 9r

This inscribed devotional community informs our understanding of Jarman’s affective aesthetic practice. In another painting, *Queer* (1992), Jarman etches the title slogan over a large heart [Figure 4]. For myself—as a consequence of being part of a generation raised in an era of rainbow capitalism and the commodification of queer histories—the painting has lost some of the immediate affective impact that it demands. Nevertheless, it is through juxtaposing this painting with a medieval counterpart that I am able to access its fuller significance. In *Chroma,* Jarman writes that: ‘red is without doubt the colour of war. The colour of life departing from a broken heart is a trickle of red blood. Sacred heart of Jesus’ (Jarman 2019b, 25). In Egerton 1821, a representation of a sacred heart follows the Passion scene, again supplemented with more dark blood drops [Figure 5]. Jarman’s use of the heart symbol as a serious representation of the sacred heart icon draws attention to the image as a sign of suffering and communal mourning. The painting situates itself in its contemporary context of interrupted ceremonies, bereavement, and oppression. But these conditions are expressed through the transhistorical invocation of affective practices. The thick impasto surface of Jarman’s work invites its viewer to imagine touching its ridges. The brushstrokes have an immediacy that makes their creator present in the object, witnessing the act of making. Similarly, the addition of new blood drops over the pasted woodcut in Egerton 1821 invite the viewer to dwell in the act of making as a means of rendering the image a living subject. These are moving traces: active, vital, emotive. The painting remains a vessel of Jarman’s touch, and in that touching quality, the viewer is implicated in a shared experience of desire and suffering. The artwork becomes a vessel of the artist’s body, emulating the affective nature of the medieval sacred image, which becomes the active subject beneath longing, stroking hands. The modern gallery forbids its visitors from performing that devotional action. The viewer here must keep their experience internal, but that interiority is no obstacle to an affective relationship between image and viewer, between us and Jarman’s blood.

## Conclusion: The holy blood of St. Derek of Dungeness

Living under the increasingly unavoidable awareness of his own mortality, Jarman observed the early stages of his transformation into queer saint, on both a literal and a cultural level. In 1991, Jarman was canonised by the Sisters of Perpetual Indulgence, an order of queer nuns, as ‘St Derek of the Order of Celluloid Knights of Dungeness,’ in recognition of his contributions to film and his other excellent attributes, such as his beautiful nose. As a medievalist, Jarman performed a learned self-identification with hagiographic traditions. Subtitling his polemic, *At Your Own Risk*, ‘A Saint’s Testament,’ he wrote proudly of being the ‘first Kentish saint since Queer Thomas of Canterbury’ (Jarman 2019a, 118). At his home, Prospect Cottage in Dungeness, Jarman set about producing assemblages inspired by medieval traditions of devotion, referring in his journals to the making of ‘a gilded canvas in the manner of a relique: a coffin nail, locks of hair, a broken comb found on my walk yesterday, a diamond, gold ring, ruby blood drop, one lucky stone hanging from a string and a pale pink condom. / Nick, Robert, Terry, Howard, David’ (Jarman 2018a, 104). Enshrined in its sacred container, the condom becomes a blood relic, positioned alongside the ruby drop as a memorialising object. The names recall Jarman’s friends, lost to AIDS. Visitors found Jarman surrounded by these icons. Following the publication of *Modern Nature* in 1991, Jarman experienced the beginnings of the transformation of his home into a pilgrimage site (Jarman 2018b, 8). Pilgrimage and relic devotion are predicated on the needful presence of the venerated object. As Julian writes of her vision of Christ: ‘he showed himself on earth thus as it were in pilgrimage: that is to say, he is here with us, leading us’ (Julian 2015, 159). Sacred travel relies on the belief in the physical, proximate existence of holy blood, not only as an idea or as a historic memory, but as a continued reality. These remains become agential. Prospect Cottage today remains a pilgrim destination for those who seek to be close to St. Derek, not simply to read his journals or watch his films, but to experience the living traces of his presence.

Reflecting on his response to devotional iconography, Jarman writes in *Modern Nature*: ‘My paintings scramble the initials IHS HIV’ (Jarman 2018a, 231). Jarman emphasises the connection between the contemporary experience of HIV/AIDS and the figure of Christ. The standard monogram for Jesus’s name overlaps with the medical acronym, the letters forming an imperfect anagram. Through this conjunction, HIV+ blood becomes holy blood, the source of salvation, desire, community and healing. The biological realities of blood-borne contagion are not forgotten—but are transcended, sublimely. Reclaiming his body from the polluting context of the 1990s, Jarman resituated his blood in medieval medically-informed discourses of queer desire and devotion. In being saintly, Jarman’s blood is rendered salvific, curative, desirable to touch. Contemplating Jarman’s blood allows those of us who seek him to find him still here with us, leading us back into the past with heightened feeling of its present value.

## Footnotes

1 TGA 20157/1/5 Jarman, Tate Britain Archives, unnumbered page.

2 I am grateful to Robert Mills, who generously shared with me a list of Jarman’s books, as catalogued by Keith Collins at the time of Jarman’s death.

3 All translations from Middle English, Latin, and Arabic into modern English are my own, except where otherwise stated.

4 TGA 20157/1/5 Jarman, Tate Britain Archives, unnumbered page.

5 TGA 20157/1/4/10 Jarman, Tate Britain Archives, p. 4.

6 TGA 20157/1/5 Jarman, Tate Britain Archives, p. 52
